# Automated Segmentation of Light-Sheet Fluorescent Imaging to Characterize Experimental Doxorubicin-Induced Cardiac Injury and Repair

**DOI:** 10.1038/s41598-017-09152-x

**Published:** 2017-08-17

**Authors:** René R. Sevag Packard, Kyung In Baek, Tyler Beebe, Nelson Jen, Yichen Ding, Feng Shi, Peng Fei, Bong Jin Kang, Po-Heng Chen, Jonathan Gau, Michael Chen, Jonathan Y. Tang, Yu-Huan Shih, Yonghe Ding, Debiao Li, Xiaolei Xu, Tzung K. Hsiai

**Affiliations:** 1 Division of Cardiology, Department of Medicine, David Geffen School of Medicine, University of California, Los Angeles, California USA; 2 Ronald Reagan UCLA Medical Center, Los Angeles, California USA; 3 Veterans Affairs West Los Angeles Medical Center, Los Angeles, California USA; 4 Department of Bioengineering, Henry Samueli School of Engineering and Applied Sciences, University of California, Los Angeles, CA USA; 5 Biomedical Imaging Research Institute, Cedars-Sinai Medical Center, Los Angeles, CA USA; 6 School of Optical and Electronic Information, Huazhong University of Science and Technology, Wuhan, China; 7 Department of Biomedical Engineering, Viterbi School of Engineering, University of Southern California, Los Angeles, CA USA; 8 Department of Biomedical Engineering, National Cheng Kung University, Tainan City, Taiwan; 9 Department of Biochemistry and Molecular Biology, Mayo Clinic College of Medicine, Rochester, MN USA; 10 Division of Cardiovascular Diseases, Mayo Clinic, Rochester, MN USA

## Abstract

This study sought to develop an automated segmentation approach based on histogram analysis of raw axial images acquired by light-sheet fluorescent imaging (LSFI) to establish rapid reconstruction of the 3-D zebrafish cardiac architecture in response to doxorubicin-induced injury and repair. Input images underwent a 4-step automated image segmentation process consisting of stationary noise removal, histogram equalization, adaptive thresholding, and image fusion followed by 3-D reconstruction. We applied this method to 3-month old zebrafish injected intraperitoneally with doxorubicin followed by LSFI at 3, 30, and 60 days post-injection. We observed an initial decrease in myocardial and endocardial cavity volumes at day 3, followed by ventricular remodeling at day 30, and recovery at day 60 (*P* < 0.05, n = 7–19). Doxorubicin-injected fish developed ventricular diastolic dysfunction and worsening global cardiac function evidenced by elevated E/A ratios and myocardial performance indexes quantified by pulsed-wave Doppler ultrasound at day 30, followed by normalization at day 60 (*P* < 0.05, n = 9–20). Treatment with the γ-secretase inhibitor, DAPT, to inhibit cleavage and release of Notch Intracellular Domain (NICD) blocked cardiac architectural regeneration and restoration of ventricular function at day 60 (*P* < 0.05, n = 6–14). Our approach provides a high-throughput model with translational implications for drug discovery and genetic modifiers of chemotherapy-induced cardiomyopathy.

## Introduction

Chemotherapy-induced cardiotoxicity remains an unmet clinical challenge with the most frequent complication being cardiomyopathy leading to heart failure^1^. In humans, injured myocardium undergoes apoptosis leading to fibrosis^1, 2^, whereas zebrafish *(Danio rerio)* possess a capacity to undergo regression in fibrosis and regeneration of myocardium in injured hearts^3^. Despite having a two-chambered heart, the zebrafish cardiac action potential and electrocardiogram (ECG) patterns are similar to those of humans^4^, thus representing a viable vertebrate model for cardiac injury and repair^3, 5, 6^. In adult zebrafish, regenerating myocardium electrically couples with uninjured myocardium^6, 7^ providing a conserved cardiomyopathy model^8^. However, the small size of the zebrafish heart hinders precise image interrogation of architecture and function.

To characterize the ultra-structural changes in doxorubicin-induced cardiomyopathy, we developed an automated segmentation approach based on histogram analysis for cardiac Light-Sheet Fluorescent Imaging (LSFI), leading to rapid image processing and 3-D reconstruction with high axial resolution and depth penetration. Currently used light microscopy techniques such as confocal microscopy are confined to imaging thin and transparent samples with background noise from out-of-focus illumination, limited depth penetration, and low axial resolution due to a large depth of field^9^. The advent of LSFI offers deep axial resolution with a large dynamic range of length and time scales, rapid data acquisition (<15 sec for adult zebrafish hearts) and reduced photobleaching via a thin sheet of laser light for selective illumination of the sample in the focal plane of the detection system^9, 10^. The application of linear scanning via single plane excitation further allows for a high resolution at low magnification with objective lenses from 1X – 10X, thereby permitting multi-scale imaging^11, 12^. In addition to the cost savings compared to high magnification optics, the low magnification lenses allow for a large working distance between the samples and illuminating/detecting lenses for non-planar imaging of live samples^13^.

Automation helps standardize imaging studies and provides a viable alternative to time-consuming manual quantification^14^. Current efforts are underway to include the use of machine learning processes to facilitate automated interpretation and improve quality control measures^15^. In this context, we developed an automated segmentation process based on histogram analysis of cardiac LSFI studies to standardize the volumetric quantitation of dynamic 3-D architectural changes occurring in experimental anthracycline-induced cardiac toxicity and repair.

To further elucidate electromechanical coupling in the injured and regenerating myocardium, we integrated micro-electrocardiogram (μECG) with high-frequency pulsed-wave (PW) Doppler to interrogate cardiac hemodynamics^16–18^. Synchronizing μECG with PW Doppler signals allowed identification of passive (early [E]-wave velocity) and active (atrial [A]-wave velocity) ventricular filling to determine E/A ratios and monitor ventricular diastolic dysfunction^6^. The PW Doppler signals further enabled us to establish the myocardial performance index by determining ventricular inflow tract isovolumic contraction and relaxation times as well as ejection times^19^.

In this context, the paralleled advances of light-sheet fluorescent imaging with automated histogram analysis and high-frequency ultrasonic transducers enabled us to unravel the 3-D architecture and electromechanical coupling of doxorubicin-induced cardiac injury and regeneration. Our results demonstrate that doxorubicin-induced cardiac injury develops ventricular remodeling, followed by activation of Notch signaling to promote cardiac regeneration and restoration of contractile function; thus, providing a high-throughput model with translational implications to drug discovery and genetic modifiers of chemotherapy-induced cardiomyopathy.

## Results

### Customized Light-Sheet Fluorescent Imaging Characteristics and Automated Histogram Analysis-Based 3-D Image Reconstruction

**Figure 1:**
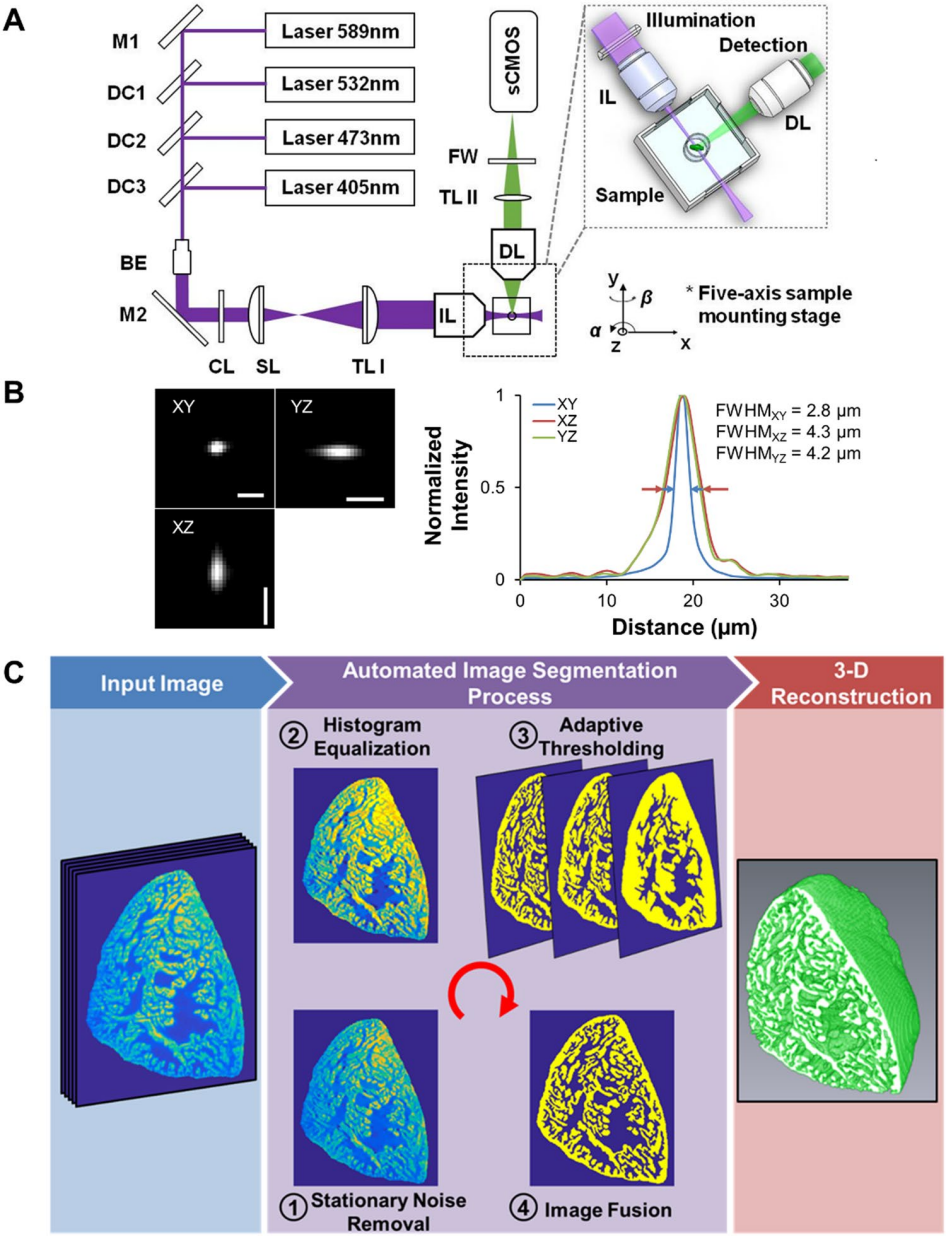
Light-Sheet Fluorescent Imaging, Histogram Image Processing, and 3-D Reconstruction. (**A**) Collimated lasers are transmitted through an illumination lens (IL) to generate a light-sheet sectioning the sample. The detection arm includes an objective lens (DL) positioned orthogonally to the illumination path for fluorescence detection. The detection axis needs to exactly conjugate the illuminated plane with the camera CMOS plane. (**B**) We obtained the lateral and axial optical spatial resolution by measuring the point spread function (PSF) of 0.5 μm beads, expressed as the FWHM from the point source in the XY, XZ, and YZ planes. (**C**) Summary of the image reconstruction and 3-D rendering process in 3 phases encompassing input image, automated image segmentation, and 3-D reconstruction. BE: beam expander. CL: cylindrical lens. DC: dichroic mirror. DL: detection lens. FW: filter wheel. FWHM: full width at half maximum. IL: illumination lens. M: mirror. sCMOS: scientific complementary metal oxide semiconductor. SL: scan lens. TL: tube lens. Scale bar: 5 μm.

Laser light is generated and reflected by a mirror following which it traverses dichroic mirrors which transmit laser light of specific wavelengths (Fig. 1A). The beam expander modulates the laser beam to become uniform. Following reflection by a second mirror, the light traverses a cylindrical lens which permits laser focusing and line pattern formation. Next, a scan lens combined with a tube lens, or known as relay lenses, modulate the beam diameter for fine-tuning. Following illumination of the sample, the orthogonally captured, non-uniform, collimated fluorescence traverses a second tube lens which focuses the beam. Subsequently, a filter wheel containing different filters permitting selection of specific wavelengths allows a particular fluorescence of interest to be detected by the digital camera. The thickness of the light-sheet on the focus is ~2 μm. When the light-sheet scanned through the heart along the Z-direction, the sCMOS camera located at the terminal end of the detection path simultaneously recorded a stack of 2-D plane images along different Z-depths. Furthermore, the high-frame rate of the sCMOS camera (up to 100 frames per second with 2048 × 2048 pixels per image) allowed completion of the entire 3-D scanning and data acquisition within tens of seconds. Thus, we demonstrate the capacity to reduce the exposure time of each image to 20 msec; thereby acquiring the entire set of raw data in <15 sec.

Raw images of the point spread function measured (PSF) using 0.5 μm beads were obtained, and the optical spatial resolution further presented as the full width at half maximum (FWHM) of the point spread function (Fig. 1B). The FWHM normalized scale on the y-axis and the distance from the camera leading to the point spread function measurement on the x-axis are depicted. The optical voxel size was 2.8 μm × 2.8 μm × 4.2 μm. The digital resolution (data not shown) is determined by the pixel size of the camera and the stepping size of the motor. We utilized a high-speed, high-resolution sCMOS camera with an XY pixel size of 6.5 μm. Using the 4X/0.13 objective/numerical aperture effectively reduces the XY pixel size to 1.625 μm. The XZ and YZ pixel sizes are a function of the digital resolution and the chosen Z-step size was set at 3 μm. As a result, the effective digital voxel size was 1.625 μm × 1.625 μm × 3 μm.

Following acquisition by LSFI, images underwent a 3-phase rendering process leading to the 3-D reconstructions of digital adult zebrafish hearts (Fig. 1C). Phase 1 involves the input of the stack of raw 2-D images. Phase 2 consists of 4 steps: Step 1 addresses stripe imaging artifacts via a stationary noise remover, Step 2 consists of a histogram equalization process to enhance contrast, Step 3 uses an adaptive thresholding algorithm to segment the myocardial tissue, and finally Step 4 fuses together the multi-scale segmented results. Phase 3 involves 3-D myocardial rendering of the segmented binary results.

To evaluate the performance of our novel automated segmentation method against an accepted reference standard, we performed blinded visual (i.e. manual) volumetric quantitation of total heart volumes, myocardial volumes, and endocardial cavity volumes in randomly chosen n = 53 zebrafish encompassing control and doxorubicin groups at days 3, 30, and 60 (Suppl. Fig. S1). Our results demonstrate a high correlation between manual and automated approaches for all volumetric quantitative analyses (Spearman *r* = 0.98–0.99, *P* < 0.0001 for all comparisons).

### 3-D Rendering of the Adult Zebrafish Heart

**Figure 2:**
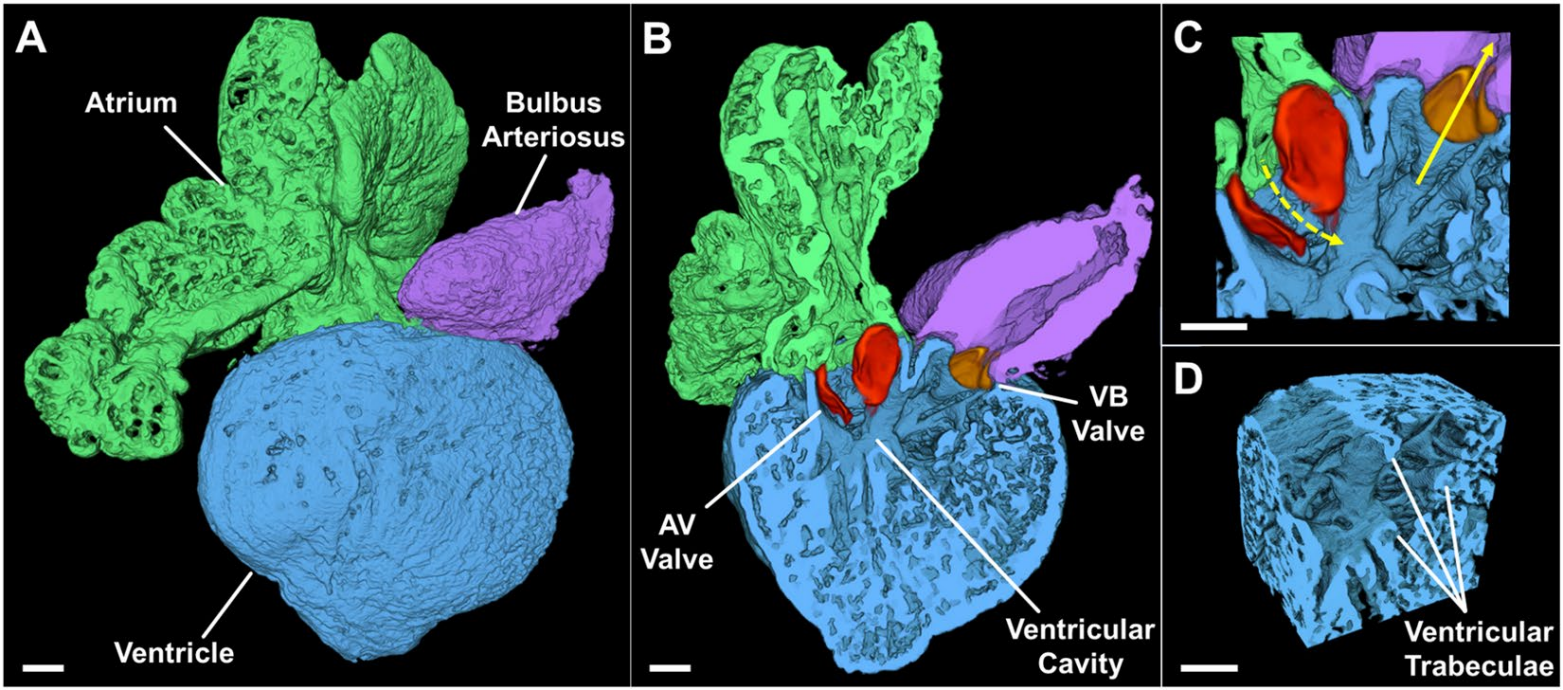
3-D Rendering of the Adult Zebrafish Heart. Example of a reconstructed heart. (**A**) The anatomic relationship of the intact atrium, ventricle, and bulbus arteriosus is shown in a surface view. (**B**) A cross-section through the atrium, ventricle, and bulbus arteriosus demonstrates the 2 leaflets of the AV valve (red) and of the VB valve (orange). (**C**) Magnification at the level of the cardiac valves showing the ventricular inflow through the AV valve (dashed arrow) and the ventricular outflow through the VB valve (solid arrow). (**D**) Magnification of a ventricular segment with highlighted trabeculae. AV: atrioventricular. VB: ventriculobulbar. Scale bar: 100 μm.

Automated segmentation permitted rapid image reconstruction of the 3-D structures of the atrium, ventricle, and bulbus arteriosus (Fig. 2A), revealing atrial and ventricular trabeculae and ultrastructure (Fig. 2B). The orthogonal ventricular inflow (dotted yellow arrow) and outflow path (solid yellow arrow) was established by the angle between the atrio-ventricular (AV) and ventriculo-bulbar (VB) valves (Fig. 2C) in the presence of highly trabeculated ventricular endocardium (Fig. 2D). Next, the automated segmentation paved the way to investigate the dynamic changes in cardiac volumes in response to doxorubicin-induced cardiac toxicity.

### Cardiac Architecture Following Doxorubicin Treatment

**Figure 3:**
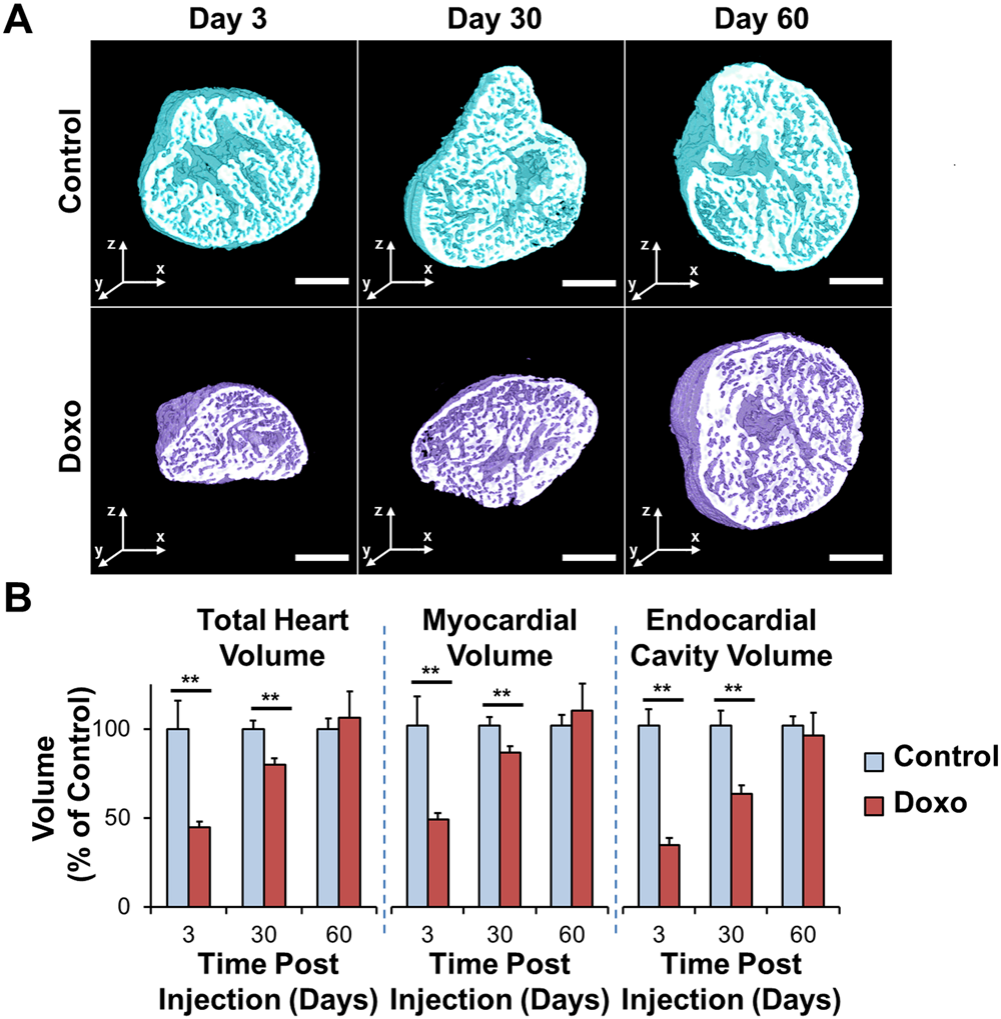
Cardiac Architecture Following Doxorubicin Treatment. Adult zebrafish hearts were harvested at days 3, 30, and 60 following treatment with doxorubicin or control vehicle. **(A)** Control hearts exhibit preserved architecture throughout the study period. In contrast, doxorubicin-treated hearts demonstrate a profound cardiac remodeling leading to acute decrease in size at day 3, followed by progressive increase at day 30, and return to control levels at day 60. **(B)** Quantitative analysis of the total heart, myocardial, and endocardial volumes normalized to control values demonstrating the cardiac regeneration process following response to chemotherapeutic injury. **P < 0.01. Doxo: doxorubicin. Scale bar: 200 μm.

The 3-D reconstruction (Fig. 3A) enabled quantitation of cardiac volumes at days 3, 30, and 60 following chemotherapy treatment (Fig. 3B for data normalized to the control reference group at each time-point, Suppl. Fig. S2 for raw data). Compared to control fish, doxorubicin treatment led to an acute decrease in myocardial and endocardial volumes at day 3 (*P* < 0.01), demonstrating global cardiac injury (Figs 3B and S2). This was followed by ventricular remodeling at day 30 (*P* < 0.01) and complete regeneration and restoration of normal architecture at day 60 (control and doxorubicin n = 7 and n = 13 at day 3, n = 19 and n = 19 at day 30, and n = 8 and n = 7 at day 60, respectively) (Figs 3B and S2). These results demonstrate the capability of LSFI combined with automated histogram analysis to monitor 3-D cardiac ultrastructural changes following chemotherapy.

### Cardiac Function Following Doxorubicin Treatment

**Figure 4:**
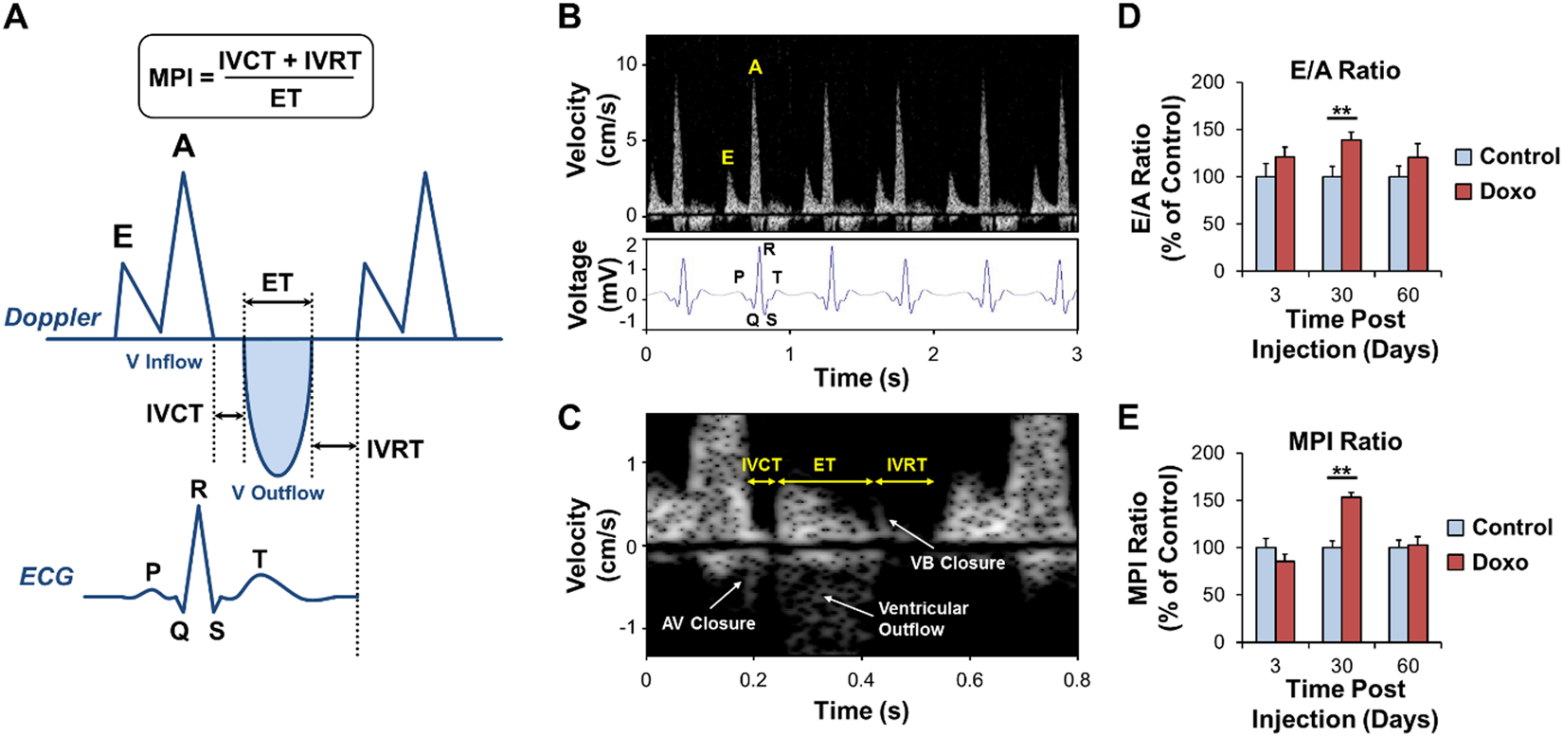
Diastolic Function and Myocardial Performance Index (MPI) by Micro-Echocardiography Following Doxorubicin Treatment. (**A**) Schematic illustration of the ventricular inflow E- and A-waves and ventricular outflow obtained by using the high-frequency ultrasonic transducer (30 MHz) for Pulsed-Wave Doppler signals. Ventricular inflow occurs during diastole when the atrioventricular (AV) valve is open, whereas ventricular outflow occurs during systole when the ventriculobulbar (VB) valve is open. A corresponding synchronous ECG signal is also illustrated, with the P-wave (or atrial depolarization) preceding the A-wave (or active ventricular filling), the QRS complex (or ventricular depolarization) preceding the onset of mechanical systole (or IVCT and ventricular outflow), and the T-wave (or ventricular repolarization) preceding the onset of mechanical diastole (or IVRT and ventricular inflow). (**B**) ECG signals were co-registered for synchronization with Doppler measurements of ventricular blood inflow E- and A-waves. (**C**) Illustration of MPI determination with IVCT, ET, and IVRT times and illustration of AV valve closure, ventricular outflow, and VB valve closure. (**D**) E/A wave ratios and (**E**) MPI parameters were measured at days 3, 30, and 60 after treatment with control vehicle or doxorubicin and normalized to control values. Following doxorubicin treatment, there was a rise in E/A ratios indicating diastolic dysfunction, as well as a rise in MPI indicating worsening of global cardiac function at day 30 followed by normalization at day 60. **P < 0.01. AV: atrioventricular. BV: bulboventricular. Doxo: doxorubicin. ECG: electrocardiogram. ET: ejection time. IVCT: isovolumic contraction time. IVRT: isovolumic relaxation time. V: ventricular. Scale bar: 200 μm.

To assess ventricular function in response to doxorubicin treatment, we synchronized micro-electrocardiogram (µECG) signals with blood flow velocities in zebrafish hearts measured using a 30 MHz ultrasound array (Fig. 4A–C). Doxorubicin-injected fish developed worsening of ventricular diastolic function illustrated by an elevation of E/A ratios at day 30 (*P* < 0.01) which normalized at day 60 (E/A ratios control and doxorubicin n = 14 and n = 18 at day 3, n = 12 and n = 20 at day 30, and n = 12 and n = 11 at day 60, respectively) (Fig. 4D for data normalized to the control reference group at each time-point, Suppl. Fig. S3A for raw data). As a corollary, doxorubicin-treated fish developed an elevated myocardial performance index – an integrated measure of both systolic and diastolic function – indicating worsening of global cardiac function at day 30 (*P* < 0.01), followed by normalization at day 60 (Figs 4E and S3B) (MPI control and doxorubicin n = 9 and n = 19 at day 3, n = 9 and n = 14 at day 30, and n = 10 and n = 11 at day 60, respectively). This myocardial performance index normalization supports that zebrafish harbor the regenerative capacity to restore chemo-induced systolic and diastolic dysfunction.

### Notch Pathway Activation Following Doxorubicin Treatment

**Figure 5:**
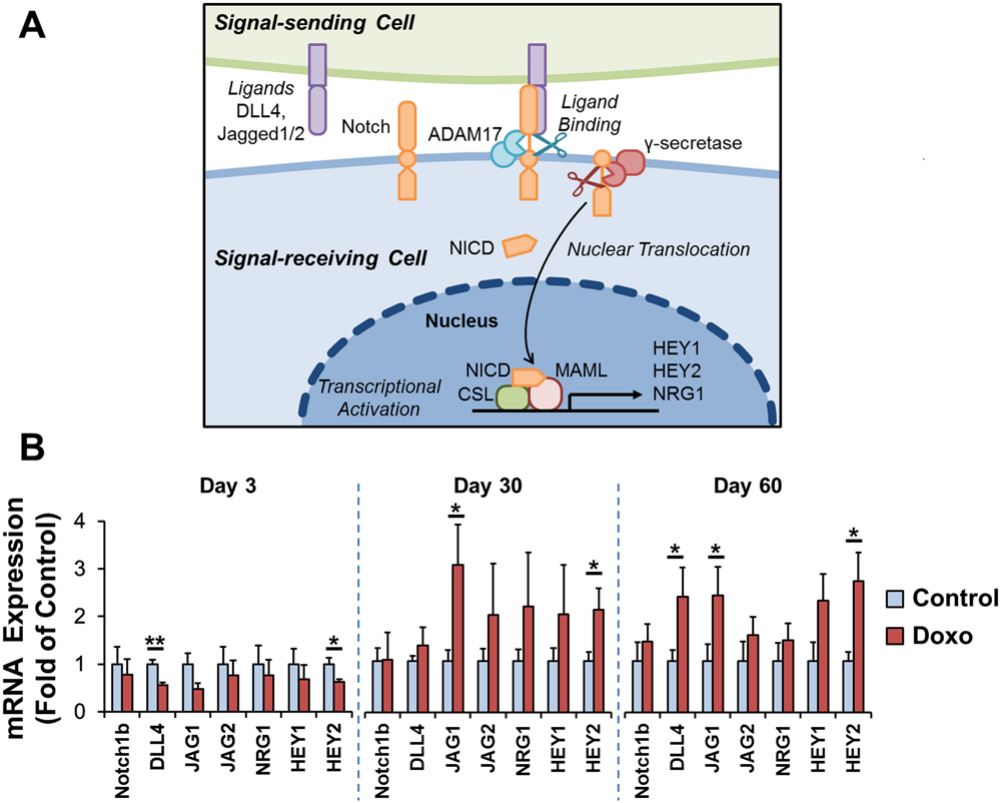
Notch Pathway Related Genes Following Doxorubicin Treatment. Key events involved in activation of the Notch pathway, implicated in cardiomyocyte proliferation and differentiation. Upon binding of ligands by a signal-sending cell, the Notch receptor gets ‘pulled’ and undergoes a conformational change. This exposes the receptor to a first proteolytic cleavage by ADAM17. Subsequently, further conformational change permits a second proteolytic cleavage by γ-secretase. This is followed by nuclear translocation of the remaining Notch intracellular domain (NICD) which leads to cooperative assembly with the transcription factor CSL and transcriptional co-activator MAML to form a transcriptional activation complex. (**B**) Zebrafish heart mRNA levels of Notch pathway related genes were determined following doxorubicin or control vehicle injection at days 3, 30, and 60 (n = 5 per condition and per time-point). There was an initial decrease of Notch pathway genes at day 3 following doxorubicin treatment, corresponding to cardiomyocyte death by activation of both apoptosis and necrosis. Thereafter, we observed increased expression of Notch pathway related genes in the doxorubicin treated group with statistical significance in the Notch ligand JAG1 and downstream effector Hey2 at day 30 and day 60 and in DLL4 at day 60. *P < 0.05, **P < 0.01. ADAM17: a disintegrin and metalloproteinase 17. CSL: CBF1, suppressor of hairless, lag-1. DLL4: delta-like ligand 4. HEY: hes related family bHLH transcription factor with YRPW motif. JAG: jagged. MAML: mastermind-like. NICD: Notch intracellular domain. NRG1: neuregulin 1.

Key components of the Notch pathway, including the 2 proteolytic activation steps via ADAM17 and γ-secretase are presented (Fig. 5A). An initial decrease in Notch pathway genes at day 3 following doxorubicin treatment was observed, supporting the notion of cardiomyocyte death by activation of both apoptosis and necrosis^1, 20^ (Fig. 5B). Subsequently, Notch-related genes were transactivated in adult zebrafish hearts, in particular the Notch ligand Jagged 1 and downstream effector HEY 2 at days 30 and 60 (*P* < 0.05). In addition to changes in cardiac architecture, these observations demonstrate an association of Notch pathways with cardiac regeneration following doxorubicin treatment. We further tested 2 additional genes not related to the Notch pathway – Axin-2 and Survivin (Suppl. Fig. S4). Axin-2 plays an important role in the regulation of the stability of β-catenin in the Wnt signaling pathway which is critical to the reparative and regenerative capacity of multiple tissues^21, 22^. Axin-2 mRNA expression was significantly upregulated at days 30 and 60 following doxorubicin treatment (*P < 0.05), consistent with myocardial regeneration in response to injury. Survivin is a multitasking protein that has dual roles in promoting cell proliferation and preventing apoptosis, in part by antagonizing caspase-3 and -7^23, 24^. Survivin mRNA expression was not significantly up-regulated following doxorubicin treatment.

### Inhibition of the Notch Pathway Abrogates Cardiac Architectural and Functional Regeneration Following Doxorubicin Treatment

**Figure 6:**
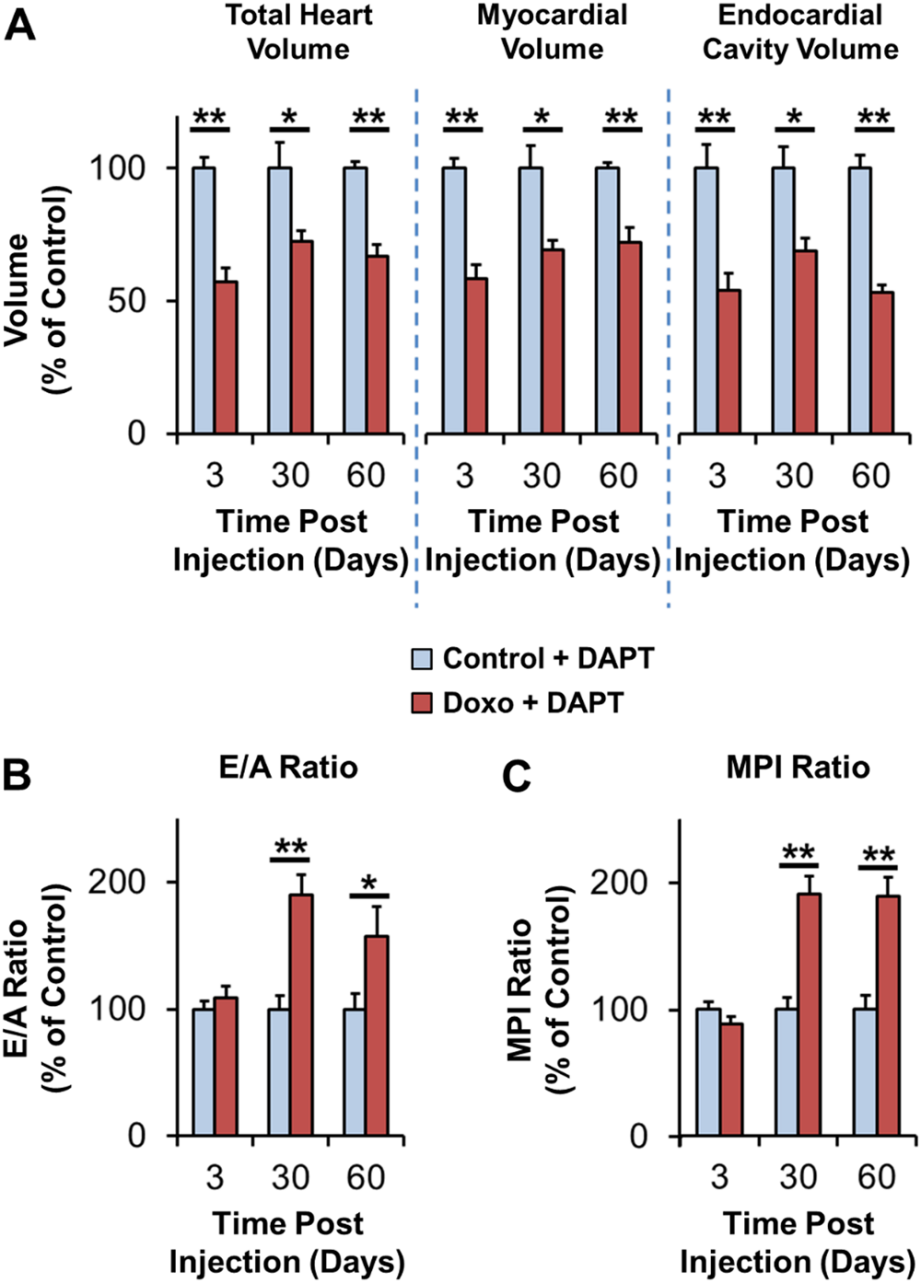
Cardiac Architecture and Function Following Doxorubicin Treatment and Inhibition of the Notch Pathway with DAPT. (**A**) Evolution of cardiac architecture following doxorubicin treatment. Following inhibition of the Notch pathway with DAPT, the heart failed to regenerate by day 60. (**B**, **C**) Evolution of cardiac function following doxorubicin treatment. Diastolic function (E/A ratios) (**B**) and combined systolic and diastolic function (MPI) (**C**) remained abnormal by day 60, indicating continued cardiac dysfunction in the presence of Notch pathway inhibition. *P < 0.05, **P < 0.01. DAPT: γ-secretase inhibitor. MPI: myocardial performance index.

To test the role of Notch signaling underlying restoration of ventricular architecture and function, we inhibited the cleavage and release of Notch intracellular domain (NICD) with the γ-secretase inhibitor DAPT following doxorubicin injection (Fig. 6A–C for data normalized to the control reference group at each time-point, Suppl. Fig. S5A–C for raw data). This led to an inability of the adult zebrafish heart to regenerate structurally, demonstrated by total heart, myocardial, and endocardial cavity volumes that remained significantly smaller than controls at day 60 (*P* < 0.01. Cardiac volumes control and doxorubicin n = 12 and n = 9 at day 3, n = 8 and n = 7 at day 30, and n = 6 and n = 6 at day 60, respectively) (Figs 6A and S5A). In parallel, ventricular diastolic dysfunction as measured by elevated E/A ratios did not normalize by day 60 (*P* < 0.05. E/A ratios control and doxorubicin n = 8 and n = 13 at day 3, n = 11 and n = 11 at day 30, and n = 7 and n = 10 at day 60, respectively) (Figs 6B and S5B). Similarly, myocardial performance indexes remained elevated, indicating persistence of impaired cardiac function post-injury when Notch signaling was blocked (*P* < 0.01. MPI control and doxorubicin n = 11 and n = 12 at day 3, n = 14 and n = 12 at day 30, and n = 8 and n = 12 at day 60, respectively) (Figs 6C and S5C). These observations demonstrate Notch-mediated cardiac regeneration and functional recovery following doxorubicin-induced injury.

## Discussion

The present body of work applies laser light-sheet fluorescent imaging with automated segmentation for analyses of 3-D cardiac architecture and ultra-structure following doxorubicin-induced toxicity. The use of E/A ratios and MPI analyses further quantified the Notch-dependent recovery of cardiac function. Thus, we establish a high-throughput imaging method with applicability for drug screening and genetic modifiers for adult zebrafish as a conserved vertebrate model of chemotherapy-induced cardiomyopathy.

Previous work by Poss *et al*. established the cardiac regenerative capacity of zebrafish hearts, by following post-surgical amputation of the ventricular apex over time^3^. The authors demonstrated that by day 60 after surgical amputation, the ventricular surface area had returned back to normal^3^. Similar results were observed following a one-time cryoinjury of the zebrafish ventricle^5, 6^. To further investigate the dynamics of architectural and functional changes, as well as involvement of molecular pathways in the cardiac regeneration following chemotherapy by day 60, we also analyzed the day 3 and 30 time-points.

Currently, there is a paucity of robust and reproducible imaging tools in the adult zebrafish model for the accurate and quantitative study of cardiac structure and function. Super-resolution microscopes known as light-sheet imaging microscopes or single plane illumination microscopes (SPIM) provide high spatial and temporal resolution, high signal-to-noise-ratio, and low level of photobleaching^10^. Whereas initially used almost exclusively in transparent tissues, primarily zebrafish and fly embryos, the advent of chemical clearing techniques have allowed for multi-scale imaging of hearts from zebrafish embryos (hundreds of microns) to adult zebrafish and neonatal mice (several millimeters)^11^. More routinely used animal imaging techniques such as micro-CT (X-ray computerized tomography) have rapid acquisition scans of a few seconds with a spatial resolution as low as 4.6 μm^25^. Micro-MRI (magnetic resonance imaging) images demonstrate superior soft tissue contrast compared to micro-CT scans, however at the expense of a lower spatial resolution (~60 μm) and poor temporal resolution (minutes to hours)^26^. Despite the development of molecular biomarker-based contrast agent development for micro-CT or -MRI^27^, these are not routinely available at present. For these reasons, both micro-CT and micro-MRI are limited from tracking fluorescently labeled zebrafish hearts for injury and regeneration. By contrast, micro-PET (positron emission tomography) imaging systems permit molecular tracking with radiolabeled immunological probes^28^, however with a spatial resolution of ~1.5 mm that remains insufficient for the zebrafish heart^29^.

Despite superior spatial resolution to track fluorescently labeled molecules of interest, confocal scanning microscopy has insufficient axial resolution, poor depth penetration, and poor temporal resolution leading to noticeable photo-damage^10^. Moreover, the 3-D image reconstruction and stitching process is laborious and not amenable to the analysis of a very large number of samples. In contrast, LSFI has a high temporal resolution (a few seconds) and near-real time 3-D image reconstruction^11^. The application of two separate sets of orthogonal lenses for illumination and fluorescence detection through selective plane excitation via laser light-sheet further reduces the photon burden to the sample, enhances the image contrast by eliminating out-of-focus contamination and reducing background noise, and improves the axial resolution under a large field-of-view^10, 11^. Limitations of optical spatial resolution are due to inhomogeneity of the light-sheet, non-uniform illumination, and scattering/diffraction of light.

Automation is increasingly available clinically across the spectrum of cardiac imaging modalities. Examples include aortic valve and plane characterization by echocardiography^30^ and cardiac CT^31^, and ventricular segmentation^32^ and border tracking^33^ by cardiac MRI. These and other studies have demonstrated the ability of automation to increase reproducibility. Contrary to a manual method of endocardial border delineation, automation increases the rapidity of data processing and 3-D image reconstruction. We developed an automated image segmentation process based on histogram analysis that led to detailed, rapid, and robust 3-D cardiac reconstruction from a stack of raw images acquired by LSFI.

In this context, we assessed the effects of doxorubicin chemotherapy on cardiac 3-D architecture by LSFI, myocardial function by high-frequency ultrasound imaging, and Notch pathway expression patterns, by determining the time-course and sequence of cardiac recovery and functional regeneration in adult zebrafish. Integrating LSFI and automated histogram analysis quantified a decrease in cardiac volumes acutely following chemotherapy, followed by remodeling and full regeneration and normalization at the 60-day experimental time-point. In parallel, micro-echocardiographic analysis demonstrated cardiac systolic and diastolic dysfunction at the 30-day post-anthracycline treatment cardiac remodeling stage, followed by subsequent recovery to normal function. Our observations are of physiologic relevance^34^, supporting an adaptation of cardiac function following exposure to the cardiotoxic agent.

Notch signaling has been previously implicated in cardiac protection following doxorubicin treatment^35^. We further assessed Notch signaling-mediated myocardial regeneration to demonstrate structural and functional recovery in response to doxorubicin-mediated injury. During development, the myocardium differentiates into 2 layers, an outer compact zone and an inner trabeculated zone. Mutations in this genetic program result in congenital heart defects^36^. Previous studies have revealed the critical role of Notch signaling in the proliferation and differentiation of cardiac trabeculation^36, 37^. Notch activation in the endocardial cell results in transcription of ephrin B2 (EPHB2), which in turn regulates neuregulin (NRG1)^36^. As a secreted factor, NRG1 signals to adjacent cells to promote their differentiation into trabecular myocytes. In this context, we determined Notch signaling pathways underlying myocardial repair in response to doxorubicin treatment and demonstrated enhanced transcriptional expression of Notch ligands (Jagged 1, Delta-like ligand 4) and downstream effectors (HEY2). HEY2 forms homo- or hetero-dimers that localize to the nucleus and interact with a histone deacetylase complex to repress transcription by cardiac transcriptional activators such as GATA4 and GATA6^38, 39^. Treatment with the γ-secretase inhibitor DAPT to inhibit the cleavage and release of NICD mitigated myocardial repair following doxorubicin-mediated injury, further corroborating the key role of Notch signaling in this recovery.

In summary, we interfaced chemical clearing-mediated light-sheet fluorescent imaging with automated histogram analysis and high-frequency PW Doppler transducers to elucidate the 3-D cardiac architecture and hemodynamics, thereby advancing multi-scale imaging approaches to tissue development, organ pathobiology, and drug discovery^40^.

## Methods

### Research Design

All animal studies were performed in compliance with the IACUC protocol approved by the UCLA Office of Animal Research. Experiments were conducted in adult 3-month old zebrafish. A one-time 5 μL injection of doxorubicin (Sigma) at a dose of 20 μg/g of body weight by intraperitoneal route (Nanofil 10 μL syringe, 34 gauge beveled needle, World Precision Instruments) or of a control vehicle (Hank’s Balanced Salt Solution) was performed at day 0. Previous reports examined the therapeutic window of doxorubicin, establishing severe (>70%) mortality within 1 week only of treatment at a dose of 50 µg/g of body weight^8^. A dose of 20 μg/g of body weight on the other hand was demonstrated to produce a more acceptable balance between a robust cardiac phenotype following anthracycline chemotherapy, however in the setting of a much lower mortality of ~30% at day 60^8^ which was replicated in the current study. At days 3, 30, and 60 following therapy, zebrafish were analyzed *in vivo* by high-frequency ultrasound or harvested under microscopic guidance, the hearts removed and chemically cleared prior to *ex vivo* light-sheet imaging, or mRNA isolated. Given the inherent variability of biological phenotypes when studying whole organisms, baseline values were set at 100% at each time-point (i.e. day 3, day 30, and day 60) to standardize reference observations. This provided a reference value at each time-point against which structural, functional, and molecular phenotypes were compared in an unbiased manner following chemotherapy treatment. Raw data is presented in the Supplements File.

### 3-D Image Acquisition by Light-Sheet Fluorescent Imaging

Imaging was performed using a custom-built light-sheet fluorescent imaging (LSFI) system in the Cardiovascular Engineering Laboratory at UCLA. The workflow of LSFI was characterized by the orthogonal optical paths and multi-dimensional reconstruction of cardiac structures with high spatial resolution (Fig. 1). The detailed optical setting is based on the Selective Plane Illumination Microscopy (SPIM) imaging modality^41^. In the plane illumination path, the combination of a cylindrical lens (focal length = 50 mm, LJ1695RM-A, Thorlabs) together with a 4X/0.13 objective (Plan Fluor, Nikon) line-focused the collimated laser beam (405 nm, 473 nm, 532 nm and 589 nm) into a hyperbolic light sheet with tunable parameters to illuminate the zebrafish heart (Fig. 1A). Images were captured using a sCMOS (Scientific Complementary Metal Oxide Semiconductor) camera (ORCA Flash 4.0 V2, Hamamatsu). Compared to conventional wide-field microscopy, the plane illumination mode of our system enables high axial resolution imaging (Fig. 1B) without disturbance from out-of-focus fluorescence. Following precise alignment of the LSFI system, a fully automated image acquisition was implemented using a customized LabVIEW program.

### 3-D Image Processing and Reconstruction

To compensate for camera under-sampling and remove image blurs, we applied a spline interpolation and iterative 3-D deconvolution to the reconstructed image stack. As a result, a 3-D ‘digital zebrafish heart’ was reconstructed in the last step to provide visualized output with high spatiotemporal resolution and high dynamic range. We used cardiac myosin light chain-green fluorescent protein (*cmlc2*-*gfp*) transgenic zebrafish to visualize the cardiac ventricle and precisely assess myocardial architectural fate and 3-D structural reorganization after chemotherapy. To quantify changes in cardiac volumes of interest, all of the raw data were processed with the proposed pipeline shown in Fig. 1C. A major confounding image artifact in light-sheet imaging is the stripe noise blocking the light that is caused by high absorption or high scattering structures (Fig. 1C Input Image). In this setting, the input image underwent a 4-step automated image segmentation process. (1) We first added a stationary noise remover to effectively depress the stripe imaging artifacts^42^. In particular, the general white noise was replaced from the denoising model by stationary noise assumptions, which could be generated by convolving white noise with given kernels in the frequency domain^43^. We employed a combination of three Gabor kernels with multiple sizes ([10, 1], [20, 1], and [10, 3]) to better estimate the stripe artifacts from the input image^44^. (2) The contrast of resulting image was further enhanced through a histogram equalization process^45^. (3) An adaptive thresholding algorithm was then followed to segment the myocardial tissue, where multi-scale local neighborhood sizes were used to cover segments with different tissue widths^46^. (4) The multi-scale segmented results were finally fused together as the end-result (Fig. 1C 3-D Reconstruction). As shown in Fig. 1C, the artifacts were largely removed, and segmentation results preserved both the large and small structures from the input image. As a quantitative comparison, the algorithm achieved a high overlapping rate of 0.9 to the manual results in 121 images from 6 hearts, measured by Dice coefficient *DR* = 2|*A* ∩ *B*|/(|*A*| + |*B*|)^47^. The segmented binary results were used to quantify 3-D myocardial volumes and to measure 3-D total heart volumes and endocardial volumes by Amira 6.1 (FEI Software). Total heart volumes were measured by bounding the segmented results and filling the endocardial cavities, while endocardial cavity volumes were calculated by taking the difference between the total heart volumes and segmented myocardial volumes (endocardial cavity volume = total heart volume − myocardial volume) (Figs 3 and 6 and S2 and S5). By myocardial volume, we define the totality of the volume occupied by cardiomyocytes, which provide contractile forces. By endocardial cavity volume, we define the ventricular cavity lined by the endocardial endothelium, which provides the physical space for circulating blood. The 3-D renderings were processed by Amira 6.1 (FEI Software). The proposed segmentation tool is a plug-in module written in Matlab (MathWorks) code to be made publicly available through the NITRC website (http://www.nitrc.org/projects/zebrafishhearts).

### Chemical Clearing

We optimized the benzyl alcohol–benzyl benzoate (BABB, also known as Murray’s clear)^48^ optical clearing protocol. Following microsurgical isolation, hearts were placed in a phosphate buffered saline 1x solution to remove retained blood, paraformaldehyde 4% for fixation, agarose 1.5% for embedding, sequential ethanol steps (40–60–80–100%) for dehydration and BABB (with a BA:BB ratio of 1:2) for lipid removal and refractive index matching.

### µECG Acquisition

The detection of µECG signals was performed via micro-needles (AD Instruments). For ECG acquisition, the working electrode was positioned closely above the ventricle between the gills while the reference electrode was placed close to the tail. The signals were amplified 10,000-fold (1700 Differential Amplifier, A-M Systems), and filtered between 0.1 and 500 Hz at a cut-off frequency of 60 Hz. Wavelet transform and thresholding techniques were used to enhance signal-to-noise ratios (Matlab, MathWorks) for the individual ECG recording^16^.

### Cardiac Functional Phenotypes via High-Frequency Ultrasonic Transducers

Sedated fish were secured on a test-bed immersed in diluted Tricaine (0.02%) for 10–15 minutes and placed ventral side facing upward (Suppl. Fig. S6). A high-frequency ultrasound array imaging system with 30 MHz 256-element linear array transducer^17, 49, 50^ was mounted on a mechanical positioner placed vertically above the ventral side of the zebrafish at a distance of ~6 mm. The µECG electrodes are customized in order to give free access to the array transducer. A trigger signal generated from the ultrasound system is sent to the µECG recording system to synchronize acquisitions. Under the guidance of B-mode imaging, a Doppler gate (window) was positioned downstream from the atrioventricular (AV) valve in the ventricular inflow region to interrogate inflow velocities. The pulse repetition frequency (PRF) for pulsed wave (PW) Doppler was set to 9.5 kHz and the estimated Doppler angle was ~0° given the blood flow of the zebrafish cardiac chambers are in the dorsal-ventral direction. PW Doppler signals were recorded for the control and doxorubicin groups for ~3 seconds, and stored for further off-line analysis using Matlab^51^. To interrogate cardiac hemodynamics, we applied µECG signals to allow identification of PW Doppler signals of passive (early [E] wave velocity) and active (atrial [A] wave velocity) ventricular filling during diastole (Fig. 4A,B). Contrary to these measures which are dependent on the angle θ of the transducer beam with the flow of blood^52^ and hence prone to variability, measurement of various temporal segments of the cardiac cycle are independent of ultrasound signal angulation. These measures – isovolumic contraction time (IVCT), ejection time (ET) across the ventriculobulbar valve, and isovolumic relaxation time (IVRT) – determine the myocardial performance index (MPI) as follows: MPI = (IVCT + IVRT)/ET (Fig. 4A,C)^19^. MPI constitutes an integrated measure of systolic and diastolic function, with increases in MPI values indicating a worsening of cardiac function.

### Notch Signaling Following Chemotherapy

Extracted hearts were grinded with RNA lysis buffer, total RNA isolated (Qiagen) and cDNA synthetized. We tested the involvement of key components of Notch pathways in our cardiomyopathy model: receptor (Notch 1b), ligands (Delta-like ligand 4, Jagged 1, Jagged 2) and downstream effectors and targets (HEY1, HEY2, and Neuregulin1). 6 zebrafish hearts were pooled for each n with a total of n = 5 per condition and per time-point and expression levels determined by quantitative RT-PCR normalized to baseline expressions at day 0 prior to treatment (Fig. 5B). The zebrafish mRNA primer sequences utilized are provided in the Suppl. Table.

### Notch Inhibition in Adult Zebrafish

Following doxorubicin IP injections, adult zebrafish were treated with the γ-secretase inhibitor DAPT (SelleckChem) at 10 μM in fish water overnight 3 times a week over 60 days (Figs 6A–C and S5A–C). The DAPT solution was made fresh at the beginning of each week and was filtered after each treatment. Ventricular function was assessed at 3, 30, and 60 days post-injection as described above.

### Statistical Analyses

All values are expressed as means ± standard errors of the mean. Statistical comparisons between 2 groups were performed using Student’s t-test and *P*-values < 0.05 considered significant.

## Electronic supplementary material

Supplementary Figures and Table
