# Supplementary material for: Automated Segmentation of Light-Sheet Fluorescent Imaging to Characterize Experimental Doxorubicin-Induced Cardiac Injury and Repair
Source: Sci Rep. 2017 Aug 17;7:8603. doi: 10.1038/s41598-017-09152-x (PMC5561066; doi:10.1038/s41598-017-09152-x)
Supplement: Supplementary file 1 — Supplementary Figures and Table [file 41598_2017_9152_MOESM1_ESM.pdf]

Supplementary Figures and Table

Automated Segmentation of Light-Sheet Fluorescent Imaging to Characterize Experimental Doxorubicin-Induced Cardiac Injury and Repair

René R. Sevag Packard[1,2,3], Kyung In Baek[4], Tyler Beebe[4], Nelson Jen[1], Yichen Ding[4], Feng Shi[5], Peng Fei[6], Bong Jin Kang[7], Po-Heng Chen[7,8], Jonathan Gau[4], Michael Chen[4], Jonathan Y. Tang[4], Yu-Huan Shih[9,10], Yonghe Ding[9,10], Debiao Li[4,5], Xiaolei Xu[9,10], Tzung K. Hsiai[1,2,3,4]

[1] Division of Cardiology, Department of Medicine, David Geffen School of Medicine, University of California, Los Angeles, California

[2] Ronald Reagan UCLA Medical Center, Los Angeles, California

[3] Veterans Affairs West Los Angeles Medical Center, Los Angeles, California

[4] Department of Bioengineering, Henry Samueli School of Engineering and Applied Sciences, University of California, Los Angeles, CA

[5] Biomedical Imaging Research Institute, Cedars-Sinai Medical Center, Los Angeles, CA

[6] School of Optical and Electronic Information, Huazhong University of Science and Technology, Wuhan, China

[7] Department of Biomedical Engineering, Viterbi School of Engineering, University of Southern California, Los Angeles, CA

[8] Department of Biomedical Engineering, National Cheng Kung University, Tainan City, Taiwan

[9] Department of Biochemistry and Molecular Biology, Mayo Clinic College of Medicine, Rochester, MN

[10] Division of Cardiovascular Diseases, Mayo Clinic, Rochester, MN

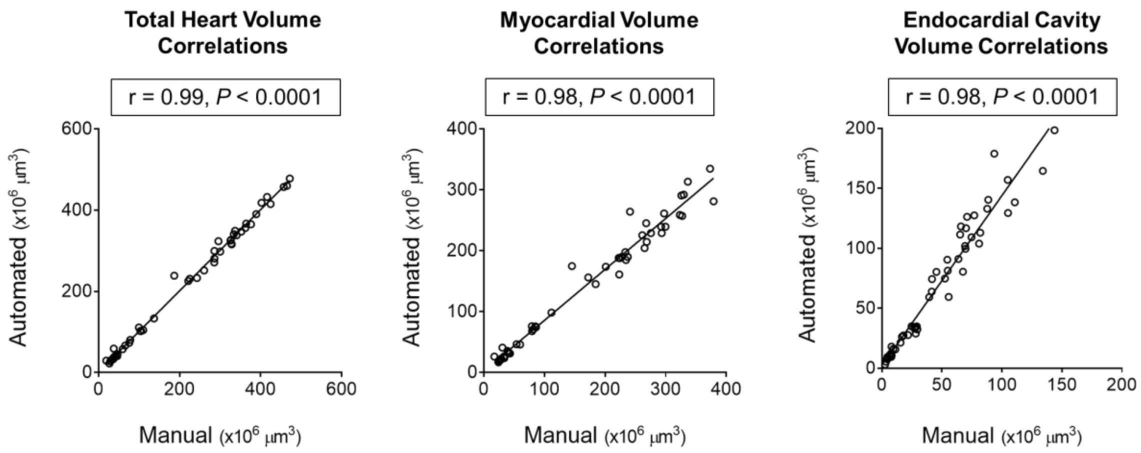

Supplementary Figure S1. Correlation of Manual and Automated Analyses for Cardiac Architecture Quantitation.

The novel automated segmentation approach based on histogram analyses was highly correlated with reference standard manual quantitation in n=53 adult zebrafish hearts encompassing control and doxorubicin groups at days 3, 30, and 60 following treatment. Spearman r=0.99 for total heart volume, r=0.98 for myocardial volume, and r=0.98 for endocardial cavity volume correlations, respectively ($P < 0.0001$ for all correlations).

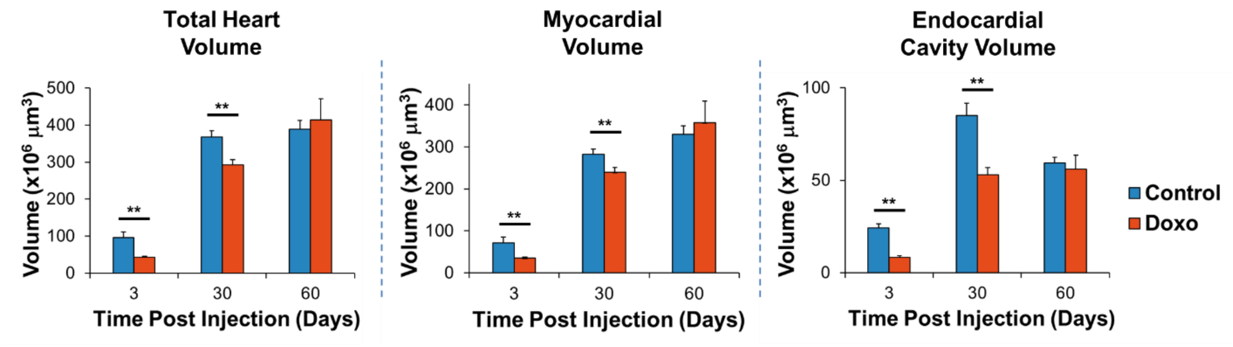

Supplementary Figure S2. Raw Data of Cardiac Architecture Following Doxorubicin Treatment.

Adult zebrafish hearts were harvested at days 3, 30, and 60 following treatment with doxorubicin or control vehicle. Raw values of the quantitative analysis of the total heart, myocardial, and endocardial volumes demonstrating the cardiac regeneration process following response to chemotherapeutic injury. **Legend.** ** $P < 0.01$. Doxo: doxorubicin.

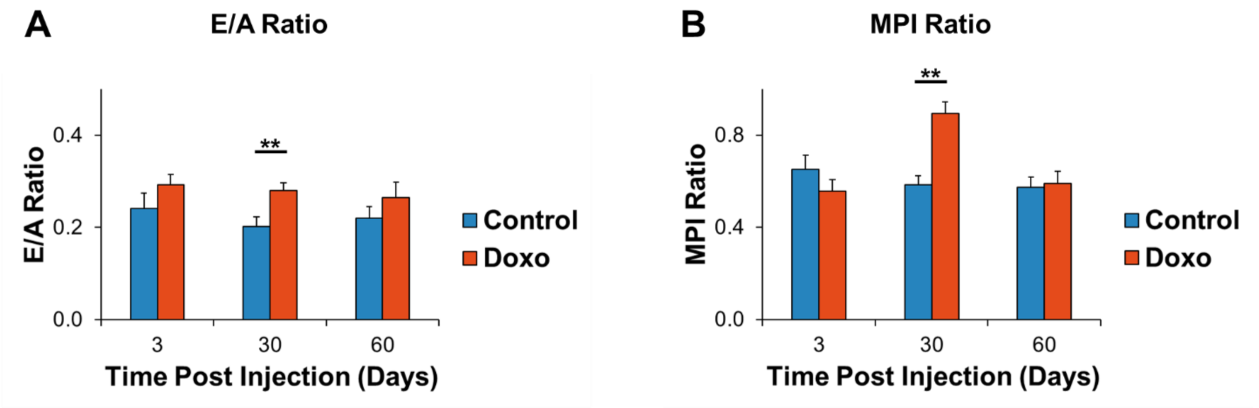

Supplementary Figure S3. Raw Data of Diastolic Function and Myocardial Performance Index (MPI) by Micro-Echocardiography Following Doxorubicin Treatment.

Raw values of **(A)** E/A wave ratios and **(B)** MPI parameters were measured at days 3, 30, and 60 after treatment with control vehicle or doxorubicin. Following doxorubicin treatment, there was a rise in E/A ratios indicating diastolic dysfunction, as well as a rise in MPI indicating worsening of global cardiac function at day 30 followed by normalization at day 60. **Legend.** ** $P < 0.01$. Doxo: doxorubicin.

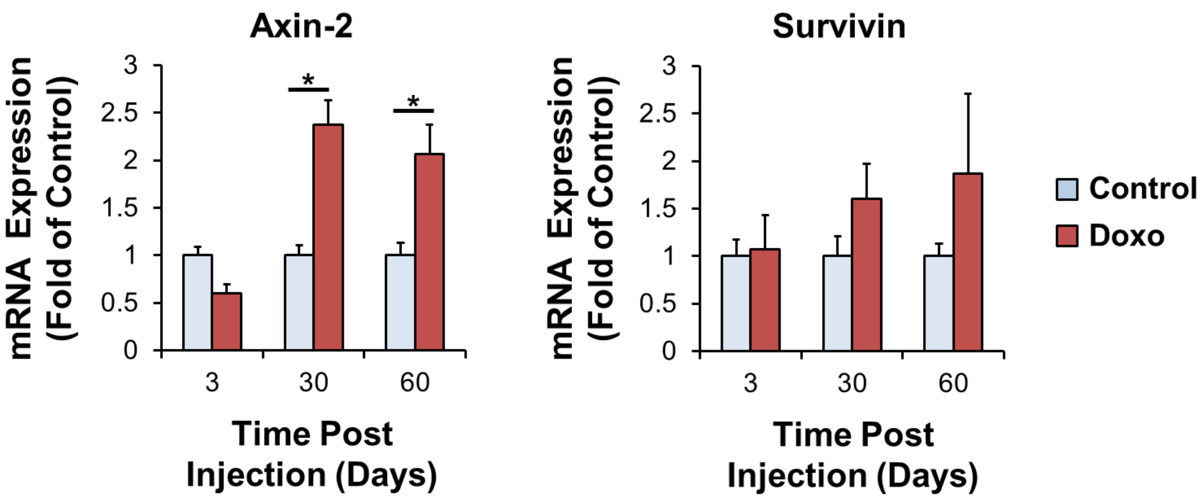

Supplementary Figure S4. Axin-2 and Survivin Gene Expression Following Doxorubicin Treatment.

Zebrafish heart mRNA levels of genes not in the Notch pathway were determined following doxorubicin or control vehicle injection at days 3, 30, and 60 (n=5 per condition and per time-point). Axin-2 expression was significantly increased at days 30 and 60 following doxorubicin treatment, whereas Survivin was not. **Legend.** * $P < 0.05$.

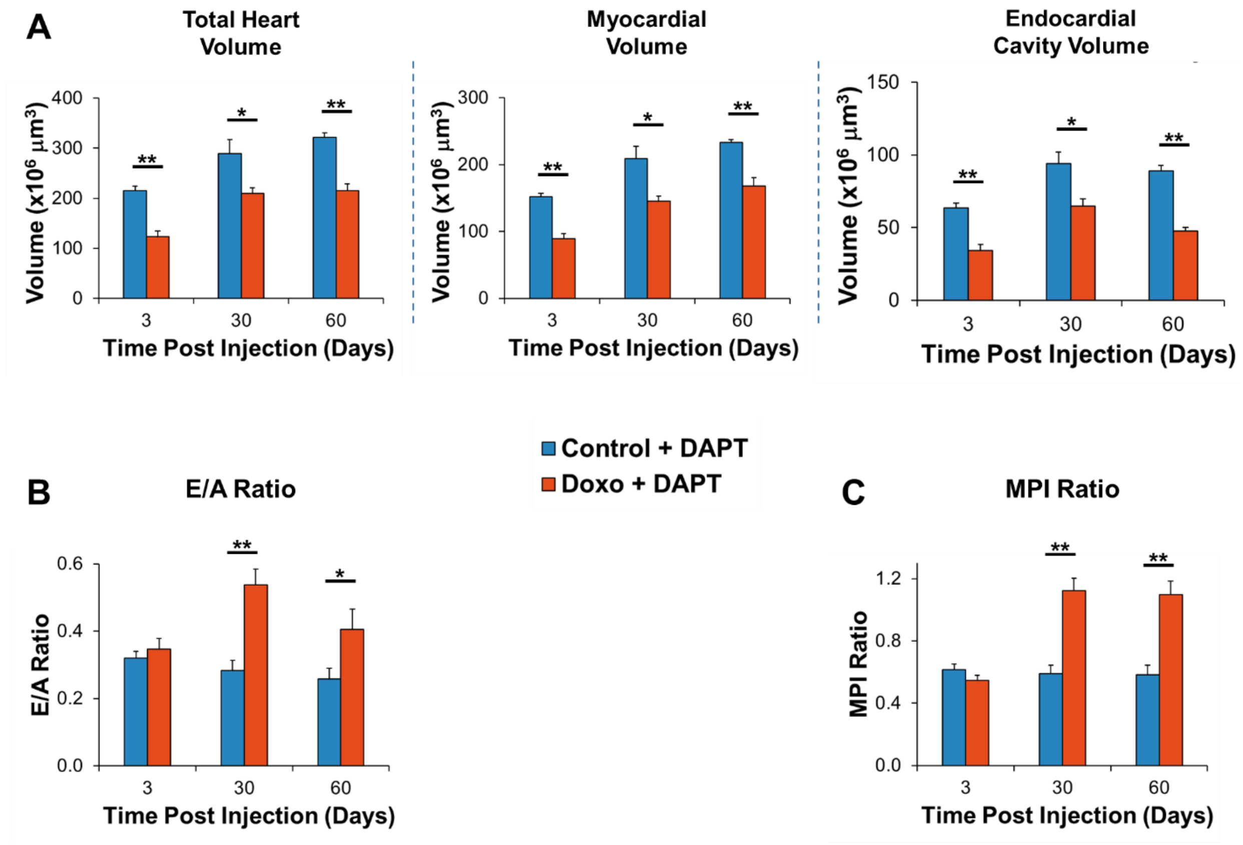

Supplementary Figure S5. Raw Data of Cardiac Architecture and Function Following Doxorubicin Treatment and Inhibition of the Notch Pathway.

(A) Raw values of the evolution of cardiac architecture following doxorubicin treatment. Following inhibition of the Notch pathway with DAPT, the heart failed to regenerate by day 60. **(B, C)** Raw values of the evolution of cardiac function following doxorubicin treatment. Diastolic function (E/A ratios) and combined systolic and diastolic function (MPI) remained abnormal by day 60, indicating continued cardiac dysfunction in the presence of Notch pathway inhibition. **Legend.** * $P < 0.05$, ** $P < 0.01$. DAPT: γ-secretase inhibitor. MPI: myocardial performance index.

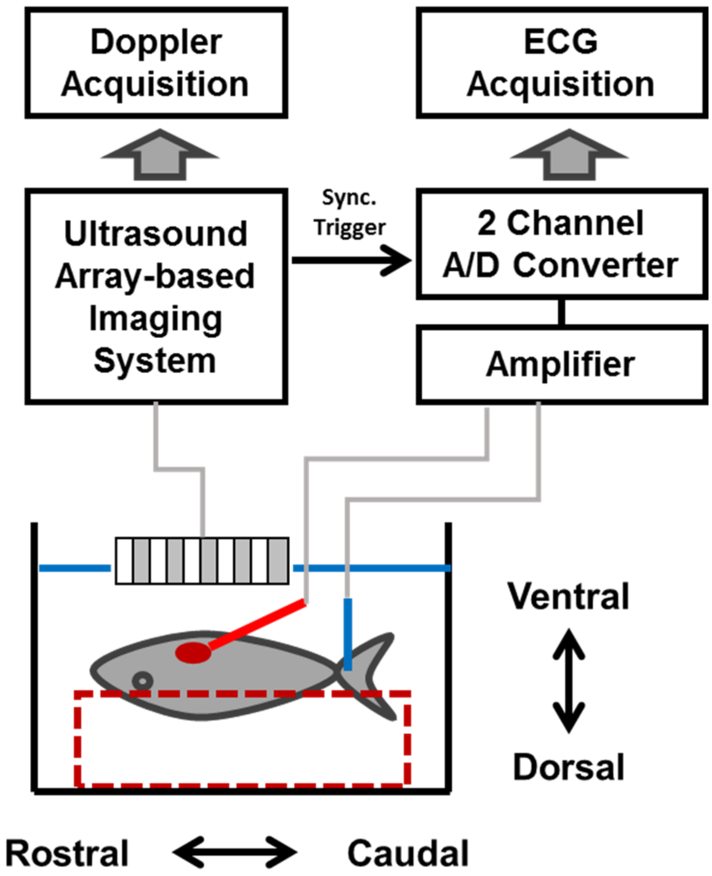

Supplementary Figure S6. High-Frequency Pulsed Wave Doppler Ultrasonic Setup.

Simultaneous measurements of μECG and Doppler signals were obtained *in vivo* in adult zebrafish hearts. **Legend.** ECG: electrocardiogram. Sync: synchronized.

**Actin**	Forward	TGGATCAGCAAGCAGGAGTACG
	Reverse	AGGAGGGCAAAGTGGTAAACGC
**Axin 2**	Forward	GGACACTTCAAGGAACAACTAC
	Reverse	CCTCATACATTGGCAGAACTG
**Delta-like ligand 4**	Forward	CAAAGTGGGAAGCAGACAGAGCTAAGG
	Reverse	CGGTCATCCCTGGGTGTGCATT
**Hey 1**	Forward	ACGATTTCAGCTCGTCGGACAGTGA
	Reverse	TCTGCGACGTTTTCTTGCTTGTACTTG
**Hey 2**	Forward	AAGATGTGGCTCACCTACAACGACATCC
	Reverse	TGGCACCAGACGACGCAACTCT
**Jagged 1**	Forward	CCGCGTATGTTTGAAGGAGTATCAGTCG
	Reverse	CAGCACGATCCGGGTTTTGTCG
**Jagged 2**	Forward	AGCCCTAGCAAAACGAGCGACG
	Reverse	GCGTGAATGTGCCGTTCGATCAA
**Neuregulin 1**	Forward	GTGTGTTTGTCCCTGTGGACGCGT
	Reverse	CCTCCTGGAGCTTCCCCTCAAACA
**Notch 1b**	Forward	CAGAGAGTGGAGGCACAGTGCAATCC
	Reverse	GCCGTCCCATTCACACTCTGCATT
**Survivin**	Forward	TCAACAAGCAAGCGAGACTCCTGCG
	Reverse	CGGCCAACCGACGAATGTTTGAAG

Supplementary Table. Primer Sequences for Quantitative Real-Time Polymerase Chain Reaction (RT-PCR) Experiments.
